# Adapting and testing a brief intervention to reduce maternal anxiety during pregnancy (ACORN): report of a feasibility randomized controlled trial

**DOI:** 10.1186/s12888-022-03737-1

**Published:** 2022-02-17

**Authors:** Heather A. O’Mahen, Paul G. Ramchandani, Dorothy X. King, Leonie Lee-Carbon, Esther L. Wilkinson, Chloe Thompson-Booth, Jennifer Ericksen, Jeannette Milgrom, Jacqueline Dunkley-Bent, Sarah L. Halligan, Pasco Fearon

**Affiliations:** 1 Mood Disorders Research Centre, University of Exeter, Perry Road, Exeter, EX4 4QG UK; 2 Centre for Mental Health, Imperial College London, 7th Floor Commonwealth Building, Hammersmith Campus, Du Cane Road, London, W12 0NN UK; 3 Central and North West London NHS Foundation Trust, Stephenson House, 75 Hampstead Road, London, NW1 2PL UK; 4 Centre for Research on Play in Education, Development, and Learning, Faculty of Education, University of Cambridge, 184 Hills Road, Cambridge, CB2 8PQ England; 5 Institute of Psychiatry, Psychology & Neuroscience, King’s College London, 16 De Crespigny Park, London, SE5 8AF UK; 6 Research Department of Clinical, Educational and Health Psychology, University College London, 1-19 Torrington Place, London, WC1E 7HB UK; 7 Essex Partnership University NHS Foundation Trust, Trust Head Office, The Lodge, Lodge Approach, Runwell, Wickford, Essex, SS11 7XX UK; 8 Parent-Infant Research Institute, Centaur Building, Heidelberg Repatriation Hospital, Austin Health, 300 Waterdale Road, Heidelberg Heights, Melbourne, VIC Australia; 9 Melbourne School of Psychological Sciences, University of Melbourne, Level 12, Redmond Barry Building, Parkville, VIC 3010 Australia; 10 NHS England, Nursing Directorate, Skipton House, 80 London Road, London, SE1 6LH UK; 11 Department of Psychology, University of Bath, Bath, BA2 7AY UK; 12 Department of Psychiatry and Mental Health, University of Cape Town, J-Block, Groote Schuur Hospital, Observatory, Cape Town, South Africa

**Keywords:** Pregnancy, Anxiety, Antenatal, Therapy, Randomised controlled trial

## Abstract

**Background:**

We investigated the acceptability and feasibility of a new brief intervention for maternal prenatal anxiety within maternity services in London and Exeter, UK.

**Methods:**

One hundred fourteen pregnant individuals attending their 12-week scan at a prenatal clinic with elevated symptoms of anxiety (GAD-7 score of ≥7) were randomly assigned to either the ACORN intervention + Treatment as usual (TAU) (*n* = 57) or to usual care only (*n* = 57). The ACORN intervention consisted of 3 2-h group sessions, led by a midwife and psychological therapist, for pregnant individuals and their partners. The intervention included psychoeducation about anxiety, strategies for problem-sovling and tolerating uncertainty during pregnancy, including communicating about these with others, and mindfulness exercises.

**Results:**

Engagement rates with ACORN met or exceeded those in primary care services in England. In the intervention arm, 77% (*n* = 44) of participants attended at least one session, 51% (*n* = 29) were adherent, defined as attending two or more sessions. Feedback was positive, and participants in the ACORN treatment group demonstrated evidence of a larger drop in their levels of anxiety than the participants in the TAU-only group (Cohen’s d = 0.42).

**Conclusion:**

The ACORN intervention was acceptable to pregnant individuals and their partners and resulted in reductions in anxiety. With further evaluation in a larger-scale trial with child outcomes, there is significant potential for large scale public health benefit.

## Introduction

For many individuals, pregnancy is a positive experience. However, anxiety during pregnancy is common. Up to half of all pregnant individuals experience distressing levels of anxiety symptoms during pregnancy [1], with 15.2% (CI 9.0–21.4) suffering diagnosed anxiety disorders [2, 3]. Anxiety during pregnancy is therefore a key mental health problem for individuals during pregnancy. Furthermore, prenatal anxiety is a strong predictor of postnatal anxiety and depression [4, 5]. There is now significant evidence from observational cohort studies showing increased rates of anxiety in individuals during pregnancy is also associated with risk of short and long term negative outcomes in their children [6]. These include higher rates of preterm delivery [7], and child outcomes such as difficult temperament [8, 9] poorer cognitive functioning [10], increased sleep problems [11] and higher rates of emotional and behavioural problems in early childhood that often continue into adolescence and early adulthood [6].

National guidelines in the United Kingdom (UK) [12], the United States [13], Canada [14], and Australia [15] have highlighted the importance of identifying and offering treatment for prenatal anxiety and depression. These guidelines have recommended routine screening for individuals as a part of pregnancy-related care, along with timely access to services for assessment and treatment. These recommendations were made despite a lack of research about the efficacy of treatments adapted for pregnancy-specific concerns on both parental and infant outcomes.

There are existing interventions outside the perinatal period that are effective in the treatment of anxiety. Cognitive Behavioural Therapy (CBT) has been subject to rigorous evaluation and has a strong evidence base for the effective treatment of anxiety disorders [16], including in a guided self-help format [17]. There is also recent evidence for mindfulness-based interventions in treating distress and worry [18]. However, recent research strongly suggests that perinatal populations have unique concerns that require adapting existing CBT and mindfulness interventions to fit the needs of perinatal individuals. In particular, pregnant individuals with anxiety report specific concerns about the health of the foetus and personal health, fear of miscarriage, fear of childbirth, worries about adapting to a new baby and anxiety about the impact of the baby on the parental relationship [19]. Such anxieties intersect in important ways with medical concerns and the inherent uncertainties of pregnancy, child birth and the transition to parenthood and may be particularly acute for individuals with past pregnancy loss or birth trauma [20]. Pregnant individuals reported these uncertainties and anxiety contributed to being both overly vigilant to troubling information, and also avoiding the development of attachment with their fetus [21–24]. Further, feelings of being overwhelmed by the physical, social and identity changes in pregnancy in the context of existing occupational and relationship stressors place pressures on pregnant individual’s problem-solving capacity [25, 26] and leave little time for self-care [27].

Rates of treatment uptake for common mental health problems during the perinatal period (15–30% [28, 29], are lower than those outside this life period (50%) [30], and part of the reason for this may be because there are few treatments adapted for anxiety during pregnancy [31]. In qualitative studies, pregnant individuals have reported a lack of willingness to engage with treatments that do not meet their immediate needs. Poor perceived treatment relevance, coupled with pregnancy-related barriers to treatment engagement, including stigma, and juggling work and numerous pregnancy-related medical appointments combine to produce poor reported treatment engagement [29, 32].

In response to this, pregnant individuals have requested that treatments take account of their practical circumstances and treatment preferences. Novel intervention approaches include delivering treatments in flexible formats [29, 32–34], including building on pregnant individuals’ reported preferences for interventions delivered in group environments which mirror antentatal course structures and provide participants with opportunities for discussions with peer and practicing skills [35]. Recent research has also demonstrated that very close (i.e., partners) and broader (friends and family) social support can impact on child development [36], via maternal prenatal and postnatal depression and anxiety [5, 37, 38]. Policy documents have likewise indicated pregnancy can be an important time period in which to engage partners [39] and have therefore recommended including partners and co-parents in prenatal care [40]. Together, research and policy suggests that antenatal anxiety interventions may benefit from the inclusion of partners within treatments.

In sum, considering the specific needs of perinatal populations is essential to address mother and their partners’ secure treatment engagement and adherence to effective treatments [33, 41, 42]. There are multiple advantages of intervening effectively in the prenatal period to reduce maternal anxiety. First, the psychological treatment is delivered in a context in which mothers are already experiencing a high level of contact with health care services and are also attending routine childbirth and parenting classes. Second, individuals may be more likely to report and receive support for anxiety symptoms in these settings [43]. Third, prenatal treatment could reduce the possible negative effects of prenatal anxiety on foetal development. Fourth, given the associations between prenatal anxiety and postnatal depression and anxiety [4, 5, 44] prenatal intervention offers the potential to prevent some episodes of postnatal depression and anxiety and improve the wellbeing of pregnant individuals, as well as reduce the potential developmental risk to the child of exposure to maternal postnatal depression and anxiety. Recent evidence on the efficacy of interventions for anxiety during the perinatal period have been mixed [45, 46] [31];, with much of the research to date focusing primarily on fear of childbirth. In an exception to this, a guided-self help intervention comprehensively adapted for perinatal individuals’s depression, anxiety and stress needs was shown to be both feasible and acceptable to participants (i.e., *Towards Parenthood* [47, 48];*.* Together, these results underline the need for further work on interventions for antenatal anxiety and suggest that the nature, content and extent of perinatal adaptations in a treatment may matter.

In this study we describe the testing of a brief intervention tailored specifically for use with pregnant individuals experiencing prenatal anxiety, based on CBT and mindfulness principles. To help ensure that the treatment was accessible and acceptable to pregnant individuals, we aimed to deliver the intervention in a group format within the context of routine childbirth/parenting courses. The group format also allowed participants to engage in social interaction and to develop a group-based identity, normalising their experiences and providing group member support for anxiety problems during pregnancy. Embedding the intervention alongside routine childbirth/parenting courses in a brief, midwife-led group format means that it has the potential to be a cost-effective mode of delivery and one that is robust to changing healthcare environments. It, therefore, has the potential to improve outcomes for a wide range of pregnant individuals, their partners and their infants, thereby reducing the overall population burden of anxiety during pregnancy.

## Aims and objectives

The purpose of this research was to assess the feasibility and acceptability of delivering a midwife and psychological practitioner-led group intervention to reduce levels of anxiety in pregnant individuals and to test key parameters relating to the feasibility of a randomised controlled trial. To this end, we conducted a feasibility randomised controlled trial (RCT) comparing the group intervention programme plus treatment as usual (TAU) to individuals receiving TAU only, and assessed key feasibility and acceptability parameters to inform the design of a future, fully powered, pragmatic RCT. The study was conducted across two diverse sites in the UK in order to ensure generalisability.

## Methods

### Study design

Working with feedback from patient and public involvement (PPI) representatives, we designed and developed a 3-session group-based intervention for anxiety, led by midwives and supported by a psychological practitioner. This drew on core CBT principles and learning from the team that developed the *Towards Parenthood* intervention [48]. Following the development of the intervention, it was piloted, then refined using participant feedback and additional input from health professionals and service users.

We conducted a feasibility RCT comparing the group intervention for prenatal anxiety plus TAU (intervention group) with TAU-only (control group). The protocol for this study has been previously published [49] and follows CONSORT [50] and SPIRIT [51] guidelines for reporting clinical trial protocols. The trial was prospectively registered, 29/10/2014, ISRCTN 95282830.

### Ethical approval and consent to participate

The trial received ethical approval from the London – Riverside National Research Ethics Service on 15 April 2014 (Research Ethics Committee reference number: 14/LO/0339). The authors assert that all procedures contributing to this work complied with the ethical standards of the relevant national and institutional committees on human experimentation and with the Helsinki Declaration of 1975, as revised in 2008. Participants were identified by a study-specific participant number in all databases [49].

### Feasibility/acceptability criteria

The primary outcome was the feasibility of the study and intervention. We did not set a priori markers for feasibility and acceptability, but accessed them against commonly used criteria. These criteria included:

1. Recruitment of 8 participants/month in 2 sites
2. Collection of data from ≥80% (trial retention)
3. Engagement of ≥70% treatment engagement [52, 53].
4. Further, we compared treatment adherence to the ACORN intervention against NHS England’s primary care mental health services (Increasing Access to Psychological Therapies; IAPT) definition (2 or more sessions; 27.5% adherence in IAPT) [54].

As a secondary marker, we also included potential efficacy as a feasibility criteria. ACORN is a brief intervention for individuals with mild-to-moderate levels of anxiety. Consistent with other, similar interventions (e.g. [53], ,we expected at least a small baseline to post-treatment between-group effect size. We also expected that the intervention effect would be sustained over the follow-up period.

### Inclusion criteria

Participants were recruited through National Health Service (NHS) prenatal scanning clinics at their 12-week scan at sites in (West) London and Exeter, UK [49]. Sites were selected for being either urban or rural and representative of the broader sociodemographics of these settings within England (i.e. racially/ethnically diverse in the London site; socioeconomically diverse and representative of well-established, rural populations in SouthWest England). Participants were either approached directly by a research assistant in the clinic, or an administrative assistant provided them with the screen and study information. Eligible participants were pregnant individuals who had no previous children, were entering their second trimester and aged 18 years and over. Pregnant individuals were invited to participate if they scored in the top quartile of scores from normed data on the Generalised Anxiety Disorder 7-item scale (GAD-7 [55]; at screening (GAD-7 = 7 or above). The GAD-7 is a 7-item self-report screening measure for general anxiety symptoms which is widely used in clinical practice and research and is validated for use in pregnancy [56].

Partners of pregnant persons who consented to take part in the study were also invited to participate in the treatment alongside the pregnant woman, the primary participant.

Potential participants were excluded if: they had insufficient understanding of English to complete the intervention or outcome measures, or they had a significant illness or disability that would make it difficult for them to participate.

### Procedures and randomisation

**Fig. 1:**
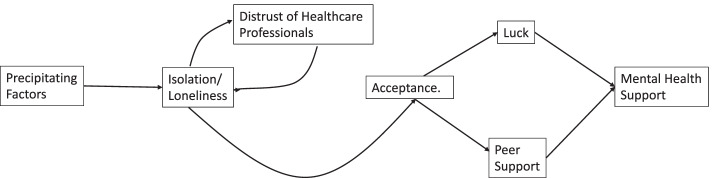
Consort Diagram

After completing the screening questionnaire for anxiety (GAD-7 [55]; and demographic questions in the prenatal scanning clinic, pregnant individuals who met eligibility criteria were invited to participate. The pregnant person’s midwife was alerted if they had a GAD-7 that was greater or equal to 7. Normal practice would have involved the midwife following up on the screen and if agreed with the woman, a referral would be made to perinatal mental health services. Upon completion of a written consent form and baseline questionnaire measures, including GAD-7 and the Edinburgh Postnatal Depression Scale (EPDS [57];, eligible participants were randomized on a 1:1 basis to the intervention or control group using a web-based computerer randomisation program, managed by a staff member in a separate research group (see Fig. 1).

Questionnaire measures were collected at baseline (T1), post-intervention (10 weeks post-randomisation T2), 8 weeks post-intervention (18 weeks post-randomisation T3), and postnatal follow up (34 weeks post-randomisation T4). Pregnant persons were paid £10 for their participation at each time-point.

### Sample size

In line with guidelines for pilot RCTs, no formal a priori power calculation was conducted [58]. A sample size of 30 participants per arm is recommended as providing sufficient data to gain an accurate estimation of the feasibility and acceptability of intervention and trial methods [59], and to provide a reasonable range of estimates of the sample size required for a definitive RCT [60]. Thus, we initially aimed to recruit 60 participants in total, although with the subsequent inclusion of a second, more rural, site (Exeter) we were able to recruit 114 participants.

### Measures

We collected a range of self-report clinical outcome measures in order to assess the acceptability of these measures and the feasibility of collecting them. The main clinical outcome measure included was for maternal anxiety:

Maternal anxiety was measured using the GAD-7 [55], a 7-item scale measuring symptoms of generalised anxiety disorder. The GAD-7 has excellent internal consistency (Cronbach’s α = .92) and good test-retest reliability (intraclass correlation = 0.83 [55];. In this study, Cronbach’s α = .82 at baseline. When used in perinatal populations the GAD-7 has yielded a sensitivity of 73.3% and specificity of 67.3%, using a cut-off score of 7 [56].

We also collected a range of other outcome measures (prospectively described in [49]. In this article we assessed means, variability, and effect sizes of measures associated with maternal wellbeing. These included the Edinburgh Postnatal Depression Scale (EPDS; Cronbach’s α = .84), a 10-item self-report scale used to assess antenatal and postnatal depression [57, 61, 62]; the 10-item pregnancy-related anxiety scale (PRAQ; Cronbach’s α = .69) [63]; and the Dyadic Adjustment Scale (DAS; Cronbach’s α = .90) [64, 65], a 32-item self-report measure of relationship adjustment. Health-related quality of life of the mother was assessed using the EuroQol-5D-3 L measure (higher scores are equivalent to poorer functioning) [66]; and health care use was measured with the Adult Service Use Schedule (AD-SUS [67];.

### Intervention

The ACORN intervention comprised a manualised group intervention, delivered as three 90-min group sessions, led by a midwife and psychological provider (e.g., trainee clinical psychologist) who attended a 3-day training in the intervention by HOM. Sessions were audiotaped and reviewed for fidelity at group supervision sessions, led by HOM. Treatment sessions were held at three-week intervals, with the aim of maintaining participant engagement, balancing participant attendance in group sessions with their medical appointments, whilst also providing participants with time to try out practical strategies in-between sessions.

The three group sessions covered key themes determined in collaboration with the research literature and our PPI group. Sessions focussed on perinatal adapted strategies to managing worry. The primary strategies were centered on problem-solving and managing uncertainty. Managing uncertainty strategies were adapted from Dugas [68] and also included mindfulness-based approaches that were acceptance and compassion-focussed (i.e., loving-kindness towards the fetus). Given the importance of social support during the perinatal period, sessions also included content on communicating about problem-solving with important others. Participants were asked to schedule soothing, self-care related activities each session. See Table 1 for details about the content of each session.

**Table 1:** Content of individual intervention sessions

Session	Focus
1	Psychoeducation on anxiety during pregnancy, taking care of yourself (rewarding activity scheduling, mindfulness-compassion).
2	Problem-solving about pregnancy-specific worries, avoidance and anxiety, Partners only: reflective listening, managing uncertainty using acceptance and distress-tolerance skills. Mindfulness-loving kindness to fetus
3	Communication with partner about pregnancy specific worries using problem-solving framework, coping with stress together, relapse prevention

All participants in the intervention arm continued to receive their usual care during pregnancy, and had access to the usually available range of interventions for prenatal anxiety and other physical and mental health problems.

### Control: treatment as usual

Participants randomised to the control group continued to receive their usual care for prenatal anxiety. There is currently no standard model of care for prenatal anxiety, however midwives are expected to screen for mood and anxiety problems and refer on to care which may include care provided by their GP, specialist mental health midwife, health visitor, or local primary or secondary care mental health team.

### Data analysis

Data analysis was primarily descriptive as this is a feasibility study. Participant flow through the study is presented following CONSORT guidelines (Fig. 1 [50];.

Analyses were conducted with SPSS version 24 and Stata Statistical Software Release 14 [69]. First, descriptive data were analysed to calculate: i) percentage of participants meeting eligibility criteria; ii) percentage entering the randomisation phase; iii) the number of sessions completed by those in the treatment arm; iv) percentage completing the outcome measures at post-treatment follow-up. Randomization checks were assessed by between -group comparisons of baseline characteristics on all measures, using χ^2^ for dichotomous variables and one-way ANOVAs for count and interval data.

Inferential statistics were by intention-to-treat. We used multilevel regression models (STATA ME package) to provide a preliminary test of the potential for the ACORN intervention to have a differential impact on anxiety (GAD-7, PRAQ), depressive symptoms (EPDS), relationship satisfaction (DAS) and quality-of-life (EQ-5D) from baseline through the follow-up periods compared to TAU. STATA ME fits multilevel regression models for a variety of distributions of the response conditional on normally distributed random effects [70]. The approach also decomposes the effects for the individual and for the group. Mixed-effects models use all available data. The MIXED procedure was used for all clinical outcomes. All model parameters for continuous outcome measures are presented here as partial standardized effects. Effects for all outcomes measures were adjusted for variance by site.

Effect sizes were standardized effects between conditions from baseline to follow-up, obtained by dividing the unstandardized estimated effects by the pooled standard deviation of the primary outcomes [71].

## Results

### Recruitment and trial adherence

Overall, 1249 pregnant individuals were screened for the study. Of these, 240 (19.2%) participants met the screening criteria and were contactable. Reasons for non-eligibility are included in the CONSORT diagram. Most participants (79%) had a GAD-7 score < 7. A further 9% of eligible individuals stated they were not interested in being part of a clinical trial, and 10% of individuals could not be contacted by the research team, despite multiple attempts at contact. Very few participants (< 1%) were already receiving a psychological intervention for their anxiety or mood, and only one person declined to be a part of the study because the intervention was offered in a group format. Of the eligible individuals, 114 (47.5%) were randomised to receive either the intervention plus TAU (*n* = 57) or TAU only (*n* = 57) (study *n* = 114; 70 recruited in the London site, and 44 in the Exeter site) (see Fig. 1 for the CONSORT diagram).

Characteristics of the participants are shown in Table 2. The mean age of the individuals participating was 31.5 years. Most (87%) were married or cohabiting with their partner. The majority ethnicity was white and the most participants had completed a university degree. There were no significant between group differences on any of the demographic or clinical variables, except baseline EPDS; pregnant individuals in the treatment condition had higher EPDS scores than those in TAU (see Table 2).

**Table 2:** Demographic characteristics of participants

Characteristics	Frequency/Distribution
Maternal Age, *M* (*SD*)	31.5 (5.09)
Race, *n* (%)	
White	72 (63.2)
Asian	11 (9.6)
Black	4 (3.5)
Multiracial	3 (2.6)
Other/ not answered	24 (21.1)
Relationship Status, *n* (%)	
Married/cohabiting	99 (86.9)
Single	12 (10.5)
Separated	2 (1.8)
Other/ not answered	1 (0.9)
Highest Educational Level, *n* (%)	
Some highschool	24 (9.6)
Highschool diploma/A level	13 (11.4)
Higher education certificate/technical	13 (11.4)
qualification	
University Degree	35 (30.7)
(Post)Graduate degree	34 (29.8)
Other/missing	8 (7.1)
EDPS status at baseline, *M* (*SD)*	13 (4.91)
“At risk” 12 or greater, *n* (%)	64 (56)

Overall 7/114 (6%) participants withdrew from the study and follow-up data was available for 96/114 (84%), 51 in TAU and 45 in the treatment condition. There were no site differences in trial adherence.

### Treatment engagement and adherence

Table 3 shows the figures for the level of engagement and attendance at the group sessions. In total, 44/57 (77%) of participants allocated to the intervention programme and 26/44 (60%) of partners attended at least one of the three intervention sessions. Consistent with England’s IAPT service definition of adherence as two or more sessions, 51% (*n* = 29/57) adhered to the intervention. Participants who were older or who were in a relationship (*M* = 1.82, *SD* = 1.15) were attended more sessions than were individuals who were not in a relationship (*M* = .57, *SD* = .79). There were no differences in treatment adherence by site (see Table 3).

**Table 3:** Frequency of intervention sessions attended by participants

Sessions attended	*N*	%
Attended 0 sessions	13	23
Attended 1 session	15	26
Attended 2 sessions	9	16
Attended 3 sessions	20	35
Total	57	100

### Effects on anxiety

**Fig. 2:**
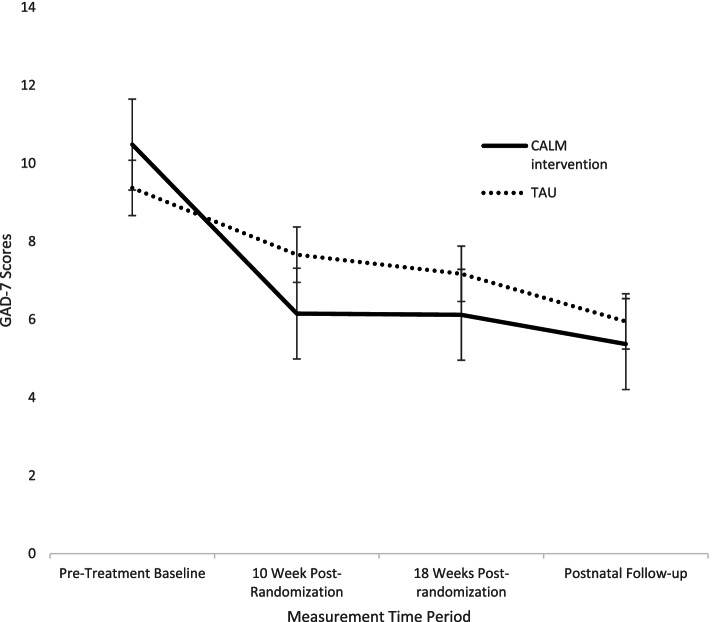
Anxiety Scores by Treatment Condition

There was a main effect of time for anxiety with both groups decreasing in anxiety scores from T1 (baseline) to each follow-up time point, T2 (10 weeks post-randomization), *t =* − 2.74, *p* < .01; T3 (18-weeks post-randomization), *t* = − 3.99, *p* < .01; T4 (34 weeks post-randomization), *t* = − 5.29, *p* < .01. This main effect was moderated by a group effect, with individuals in the treatment group having an overall greater decrease in anxiety symptoms across the follow-up period than individuals in TAU, *b* = .13, SE (.41), 95% CI (.00, 70.75). Examining change from baseline to each follow-up time point, there was a non-significant trend for a greater decline in scores in the treatment group compared to those in TAU at T2 (10 weeks post-randomization (*p* = .06), effect size = −.42 (95% CI .004 to .81) (see Table 4 for means at each time point by group) [71]. There were no other significant differences between groups at the other time points (see Fig. 2).

**Table 4:** ACORN Clinical Outcomes: Means, Standard Deviations and Group Differences Across Follow-up Periods

Measure	Condition	Baseline	10 Weeks Post Randomization	*t*	18 Weeks Post Randomization	*t*	34 Weeks Post Randomization	*t* ^1^
GAD-7	χ^2^ = 89.38*** TAU	9.53 (4.52)	7.58 (4.72)	−1.89*	6.64 (5.43)	−1.37	5.74 (4.86)	−1.26
	Treatment	10.28 (4.41)	6.40 (3.75)		5.97 (4.31)		5.20 (4.17)	
EPDS	χ^2^ = 87.26*** TAU	11.39 (5.15)	10.51 (5.23)	−2.29**	9.52 (5.33)	−2.25**	8.44 (5.00)	−2.68***
	Treatment	13.56 (4.52)	10.37 (4.34)		9.37 (4.63)		7.86 (4.91)	
PRAQ	χ^2^ = 25.76*** TAU	25.98 (4.1)	24.66 (4.2)	−.13	23.65 (4.7)	−22.89***	n/a	
	Treatment	25.67 (5.4)	22.99 (3.9)		23.19 (5.2)		n/a	
DAS	χ^2^ = 6.72 TAU	120.33 (15.4)	120.91 (20.0)	−.35	118.72 (16.8)	.32	115.89 (22.3)	.72
	Treatment	118.97 (16.8)	118.35 (23.3)		118.74 (23.3)		117.06 (20.3)	
EQ5D	χ^2^ = 1876.27*** TAU	6.60 (1.32)	6.9 (1.63)	−1.23	7.03 (1.86)	.30	5.84 (1.11)	.38
	Treatment	6.97 (1.43)	7.2 (1.21)		7.49 (1.63)		6.32 (1.45)	

*Note.* EPDS (Edinburgh Postnatal Depression Scale; Cox, Holden, & Sagovsky, 1987), PRA (Pregnancy-Related Anxiety scale; Rini, Dunkel-Schetter, Wadhwa, & Sandman, 1999); DAS (Dyadic Adjustment Scale; Spanier, 1976), EQ5D (EuroQol-5D-3 L measure; EuroQol, 1990)

***p < 0.01

**p < 0.05

* *p* < 0.1

### Effects on secondary measures

Results for the secondary measures are presented in Table 4. There were significant between group differences in depression symptoms (EPDS) at each time point; scores decreased more in the treatment group relative to TAU. At 10 weeks post-randomization the effect size was small (−.24, 95% CI −.61 to .13). These results should be interpreted with caution as individuals in the treatment group had significantly higher EPDS scores at baseline than those in TAU.

Pregnancy related anxiety scores (PRAQ) decreased significantly across the follow-up periods for both groups, but at Time 3 (18 weeks post-randomization) the rate of decline was statistically greater in the treatment group than TAU (effect size = −.28 95% CI .07 to −.46). There was no significant change in relationship satisfaction scores (DAS) scores across the follow-up period for either the treatment or TAU groups (effect size = −.03, 95% CI −.39 to .34). Although health related quality of life (EQ5D) changed between follow-up times points, there were no significant between group differences at any time point (effect size = 0.13, 95% CI −.23 to .50).

### Health care utilisation

In the intervention arm, pregnant individuals reported at time 2 (10 weeks post-randomization) that 27/44 (61%) attended General Practitioner (GP) appointments (for any reason, mental or physical health related), 35/44 (80%) saw a midwife, 3/44 (7%), a health visitor, and 13/44 (30%) saw a nurse. In addition, a small number had received therapy/counselling outside the study 8/44 (18%) or had seen a psychiatrist 1/44 (2%).

Usual care reported by participants in the TAU arm of the trial at follow-up, included 29/51 (57%) pregnant individuals having seen a GP, 47/51 (92%) a midwife, 5/51 (10%) a health visitor, and 7/51 (14%) a nurse. A small number had received therapy/counselling 6/51 (12%) or had seen a psychiatrist 2/51 (4%).

Where there were at least 5 or more observations per cell, chi-square analyses were conducted to assess differences in healthcare utilisation between groups. There were no between group differences in whether participants had seen a GP χ^2^ = .20, *p* = .66; or nurse χ^2^ = 3.56, *p* = .06; or whether they had seen a therapist or counsellor, χ^2^ = .77, *p* = .38.

Of the 91 participants who completed measures of their childbirth at the postnatal follow-up, 21/51 (42%) in TAU and 15/32 (47%) in the treatment condition reported they either had a forceps/vacuum delivery or unplanned c-section, χ^2^ = 1.3, *p* = .73.

### Treatment adherence and clinical outcomes

Numbers of sessions attended did not correlate with the primary outcome, GAD-7, *r* (1) = − .20, (95% CI: −.38, .00), nor with the depression (EPDS), *r* (1) = − .16 (95% CI: −.35, .04), EQ-5D *r* (1) = .02 (95% CI: −.18, .22), or couple functioning (DAS), *r* (1) = − .01 (95% CI: −.21, .20),. At 10-week follow-up it was, however, negatively correlated with pregnancy specific anxiety (PRAQ), *r* (1) = − .30 (95% CI: −.48, −.11).

## Discussion

We found that a brief 3-session group-based intervention met our feasibility and acceptability targets for a considerable proportion of pregnant individuals who had clinically elevated anxiety and their partners. Consistent with rates of treatment engagement in other studies [52, 53], treatment engagement in this trial was 77%. Rates of treatment adherence to the intervention (51%) compared favourably to those achieved in primary care mental health in England (27.5%; Improving Access to Psychological Therapy; IAPT) [54], and 60% of their partners attended at least one session. These results are notable, as participants were not paid for their treatment participation. They suggest that there may be potential benefits to engagement and adherence by adapting the content and delivery of interventions for general anxiety for pregnancy specific concerns. We also note that the active outreach the group supporters took (i.e., calling/texting when appointments were missed and emailing missed content) when individuals missed sessions may have also improved treatment adherence.

Further, we found that it was feasible to screen pregnant individuals for anxiety using a brief questionnaire and recruit them into intervention research in the context of their routine antenatal scanning appointments. As high rates of pregnant individuals attend their scanning appointments, our approach helped to ensure we were able to efficiently screen and offer the opportunity to participate in a study to a large number of pregnant individuals. Although scanning clinics are busy, and it was not always possible to approach or record all pregnant persons in the clinic, it provided a systematic screening method that did not rely on different healthcare provider’s willingness to screen pregnant individuals and/or refer them on to the study. This may be important in terms of knowing how to reach pregnant individuals, as other recent studies of treatments for depression and anxiety during pregnancy have reported difficulties in recruitment [45, 72]. Our study completion rates (83% at post-treatment follow-up) compare favourably with other trials conducted both during and outside the perinatal period [47, 68, 73] and demonstrate that a randomised controlled trial can work in this context, in two different geographical settings (urban: London and rural: Exeter).

Our findings also suggest that the ACORN intervention may be beneficial for treating both general and pregnancy-related anxiety during pregnancy. The results showed reductions in pregnant individuals’ general anxiety across the follow-up time periods compared with TAU alone. We note that when examining group differences at specific follow-up time periods, the greatest group difference was at 10 weeks post-randomisation and was not statistically significant at this point. This may be in part due to the fact that our cut-off was 7 or greater, and that there may have been floor effects in detecting changes in anxiety in women with milder anxiety. There were also relatively larger reductions in pregnancy-related anxiety across the follow-up period in the treatment group relative to TAU alone, with statistically significant differences between groups at 18-weeks post-randomization. Although there were moderately wide confidence intervals, the estimated effect sizes for the intervention are consistent with other psychological interventions conducted during the perinatal period [48, 74]. We suggest that future versions of the intervention may improve on the intervention tested here by building on the pregnancy adapted-material. Therapists and participants fed back informally that the ‘tolerating uncertainty’ material was helpful and highly related to participant’s pregnancy-specific concerns (e.g., fear of losing baby/childbirth), but participants stated they would like more time to focus on this material. An additional session(s) focussed on this material may therefore further improve the impact of the intervention on pregnancy-specific anxiety. Supporting this, participants who attended more sessions had lower pregnancy-related anxiety scores at 10-weeks post-randomisation. Taken together, these results, and the fact that the ACORN intervention was brief, required relatively little therapist time, and could be integrated into regular antenatal (i.e., childbirth) course care pathways, suggests that the ACORN intervention could be a promising and feasible intervention that may help to reduce the public health burden associated with maternal anxiety in pregnancy.

There are several features of the study and intervention of note. First, this intervention was developed for and focussed on treating general anxiety symptoms and pregnancy-specific worries. Although the strategies may also be useful for comorbid depression, and we saw statistically significant differences on depression, these results should be interpreted with caution as there were baseline differences in depression levels between the intervention and TAU groups. Improving the impact of the intervention on depression symptoms may involve increasing the focus/number of the sessions or also recruiting on the basis of depressive symptoms, as we may may have had floor effects on depression symptoms. We also saw little change in relationship satisfaction. This may have been due to the fact that the baseline means for relationship satisfaction were already above the norms for happy community couples [64]. We note that, consistent with the broader literature [75], ,relationship satisfaction declined across the transition to parenthood. The ACORN group did not appear to prevent declines in relationship satisfaction. It is possible that the measure used in this study (DAS) did not adequately capture components addressed in the intervention (e.g., reducing conflict). Alternatively, greater involvement from partners in the intervention (> 1 session) may have resulted in bigger impacts on relationship satisfaction, or enhanced treatment components on upholding good relationships with close others during this period may have resulted in greater benefits to the close relationships.

Second, as this was designed as a feasibility study, we are not able to provide conclusive evidence of the efficacy or otherwise of the ACORN intervention. Although we recruited a larger sample than anticipated, and found some preliminary evidence of possible benefit of the intervention for participant’s general anxiety scores and a small effect on pregnancy-specific anxiety scores, a definitive assessment will require a fully powered randomised controlled trial. We have shown that such a trial is feasible and established key methods by which it could be conducted.

Third, we used a brief screening questionnaire (GAD-7) as our main measure of anxiety. The GAD-7 is widely used and acceptable in routine clinical practice. However, it gives an indication of anxiety symptoms and does not provide a diagnostic assessment.

Fourth, we had originally designed the intervention to be delivered to each group by two trained midwives. This was not possible in the services we worked with, which were only able to release one midwife to support the group. We therefore adapted the group to be led with a midwife and mental health provider combination. This approach fits well with joint models of group delivery occurring within England’s primary care mental health (Improving Access to Psychological Treatment: IAPT) services and the NHS’ Long Term Plan newly proposed Maternity Mental Health Services, which would provide service structures that could integrate maternity and specialist perinatal mental health services. It also fits well with models of joint provider models of care in obstetrics and primary care clinics in North America, so this approach could be a potentially attractive model of delivery. Nevertheless, it does require coordination between midwifery/obstetrics and mental health services.

Finally, the intervention in this pilot was limited to individuals who spoke and read English, and most of the participants were white, which limits the generalisability of the group to a more diverse range of individuals. Also, although rate of partner participation was high, most partners were only able to join 1 session. It may be possible to extend the model to include mixed-language groups with interpreter support, or be offered in languages other than English where there are sufficient numbers within the community. Working with local ethnically diverse communities and including providers of multiracial ethnic backgrounds may help to improve uptake of the intervention. Offering the treatment using blended (in-person and remote) delivery approaches may further support participation for those with fatigue, travel and financial barriers, struggle with stigma, or who are stretched for time for mental health treatment.

## Conclusion

The findings from this study indicate that a brief group intervention for anxiety during pregnancy demonstrates acceptability to pregnant individuals and their partners, and that is feasible to incorporate this brief intervention into routine NHS practice. We were able to effectively recruit to target pregnant individuals with raised levels of anxiety in two separate sites. The programme was also associated with a reduction in levels of anxiety in pregnant participants.

With the right training in place, this intervention could easily be rolled out to enable a large number of pregnant persons to receive it. We would recommend that a further larger-scale randomised controlled trial be conducted to formally test the effectiveness and cost-effectiveness of the intervention in everyday practice. There is significant potential for long-term benefit for pregnant individuals, their partners and their children [48].

## Data Availability

The datasets generated and/or analysed during the current study are not publicly available due to limitations of ethical approval involving the patient data and anonymity but are available from the corresponding author on reasonable request.
